# Identification of Small Molecules with Type I Interferon Inducing Properties by High-Throughput Screening

**DOI:** 10.1371/journal.pone.0049049

**Published:** 2012-11-07

**Authors:** Luis Martínez-Gil, Juan Ayllon, Mila Brum Ortigoza, Adolfo García-Sastre, Megan L. Shaw, Peter Palese

**Affiliations:** 1 Department of Microbiology, Mount Sinai School of Medicine, New York, New York, United States of America; 2 Institute of Global Health and Emerging Pathogens, Mount Sinai School of Medicine, New York, New York, United States of America; 3 Department of Medicine, Division of Infectious Diseases, Mount Sinai School of Medicine, New York, New York, United States of America; The Scripps Research Institute and Sorrento Therapeutics, Inc., United States of America

## Abstract

The continuous emergence of virus that are resistant to current anti-viral drugs, combined with the introduction of new viral pathogens for which no therapeutics are available, creates an urgent need for the development of novel broad spectrum antivirals. Type I interferon (IFN) can, by modulating the cellular expression profile, stimulate a non-specific antiviral state. The antiviral and adjuvant properties of IFN have been extensively demonstrated; however, its clinical application has been so far limited. We have developed a human cell-based assay that monitors IFN-β production for use in a high throughput screen. Using this assay we screened 94,398 small molecules and identified 18 compounds with IFN-inducing properties. Among these, 3 small molecules (C3, E51 and L56) showed activity not only in human but also in murine and canine derived cells. We further characterized C3 and showed that this molecule is capable of stimulating an anti-viral state in human-derived lung epithelial cells. Furthermore, the IFN-induction by C3 is not diminished by the presence of influenza A virus NS1 protein or hepatitis C virus NS3/4A protease, which make this molecule an interesting candidate for the development of a new type of broad-spectrum antiviral. In addition, the IFN-inducing properties of C3 also suggest its potential use as vaccine adjuvant.

## Introduction

The innate immune system is the host’s early defense mechanism against invading pathogens. It functions to alert specialized cells of the presence of a pathogen and also to stall the infection until the adaptive immune system is active. Interferon (IFN) is a key regulator of the innate immune response. The potential of this cytokine (or family of cytokines) as a broad spectrum antiviral was quickly realized after its discovery in 1957 and IFN has since been used for treating infectious diseases [1], [2]. Aside from IFN, there have also been many attempts to use synthetic IFN-inducers such as polyinosinic:polycytidylic acid (poly I:C), as antivirals [3]–[6]. The role of Type I IFN (IFN-α/β) is not restricted to the induction of an antiviral-state. Among other processes, it participates in maturation and trafficking of dendritic cells (DC), T cell cross-priming and, stimulation of humoral immunity or isotype switching [7]. These are all characteristics that are highly desirable in a vaccine adjuvant and thus IFN and IFN-inducing molecules have also been extensively explored as vaccine adjuvants [8]–[10]. Any molecule with the ability to modulate the innate immune system by promoting the synthesis of IFN would have potentially dual application as both an antiviral and a vaccine adjuvant.

The induction of IFN starts with the recognition of a danger signal. Several receptors, known as pattern recognition receptors (PRR), are involved in the identification of different molecular patterns generated during intracellular pathogen invasion or cellular stress situation within the cell. The most studied PRR are the so called Toll-Like Receptors (TLR). These are Type I integral membrane proteins located in the plasma membrane and in several of the internal membranous compartments such as endosomes or lysosomes. TLRs recognize a wide variety of ligands including bacterial peptidoglycan (TLR1, 2 and 6), double stranded RNA (TLR3), DNA (TLR9), single stranded RNA (TLR7 and 8) or lipopolysaccharide (TLR4). Activation of TLRs triggers a signaling cascade that result in the activation of the AP-1, NFkB and IRF transcription factors and ultimately in the production of type I IFN. In the past, great efforts have been made to obtain synthetic TLR agonists. In fact, several TLR agonists are being explored clinically for treatment of viral infections [5], [6], [11]–[13], with varied degrees of success depending on the disease, the nature of the molecule, the dose and the route of administration.

**Figure 1:**
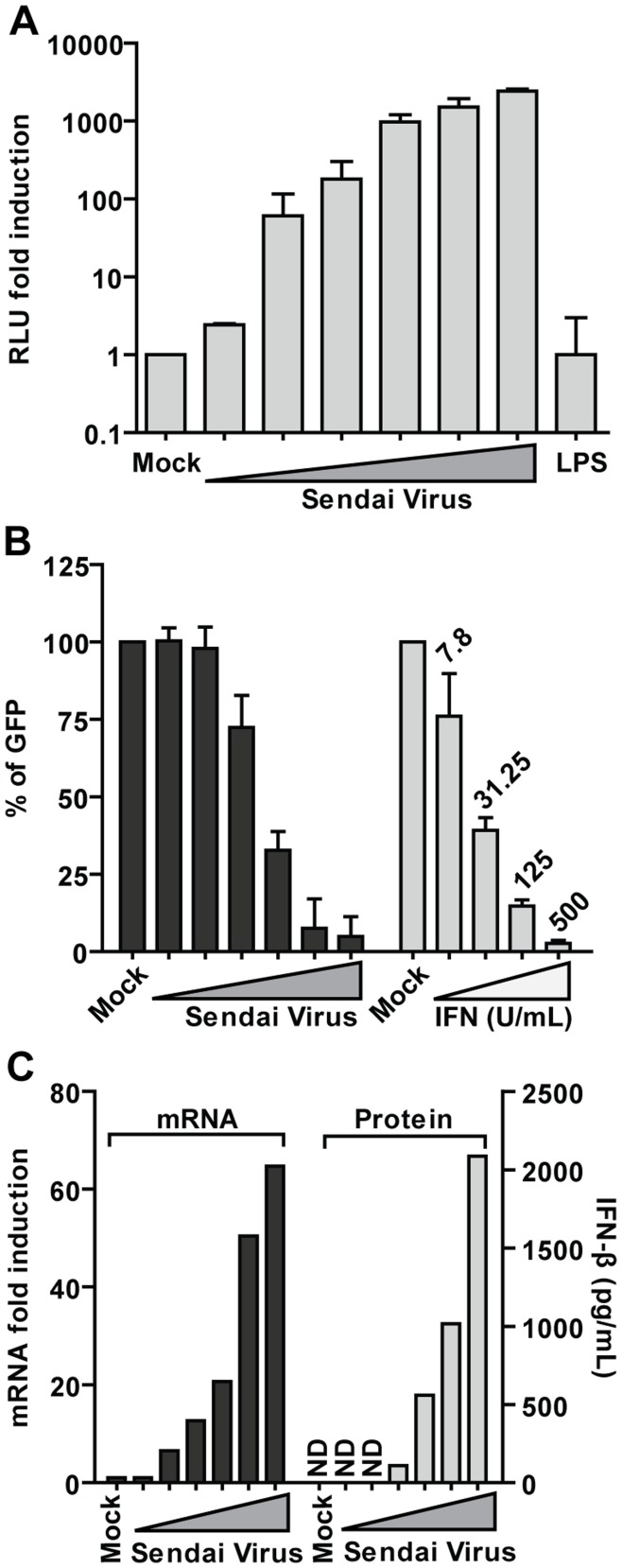
Characterization of the 293T-FF reporter cell line. 293T-FF cells were stimulated with increasing amounts of DI-rich Sendai virus Cantell or lipopolysaccharide (LPS, 100 µg/mL) as a negative control. Twenty-four hrs later the media was removed and the luciferase signal (A) or mRNA (C) measured from the cells. Supernatants were collected and the secreted IFN-β measured by ELISA (C) or in a VSV-GFP bioassay (B). Fresh media containing either 0, 7.8, 31.25, 125 or 500 U/mL of universal IFN was used as a positive control in the bioassay. Stimulation for all the panels was done at the same time and in the same format and consequently the same amount of Sendai was use for each data point across the different panels. As a result, there is a direct correlation between panels A, B and C that allows to estimate, for a given value of luciferase, the IFN-β mRNA induction, the IFN-β protein levels or the protection against VSV-GFP in a viral bioassay in 293T-FF cells. **A.** Luciferase (measured as Relative Luminescence Units, RLU) fold induction after treatment with Sendai virus or LPS over the mock treated cells. **B.** Percentage of green fluorescent protein signal (GFP), as an estimation of VSV-GFP replication, at 24 hrs after infection. The fluorescence obtained from mock-treated cells was set to 100%. **C.** IFN-β mRNA fold induction over mock treated cells (left) and IFN-β secreted protein (pg/mL) (right) from 293T-FF measured by qRT-PCR and ELISA respectively. ND indicates not detectable levels. Error bars represent standard deviation of three replicates.

A novel group of receptors that can elicit IFN synthesis, particularly in response to RNA viruses, is the RIG-I-like receptor (RLR) family. This group consists of three members, the Retinoic acid-Inducible Gene 1 protein (RIG-I), the Melanoma Differentiation-Associated protein 5 (MDA5), and the Laboratory of Genetics and Physiology protein 2 (LGP2), all located in the cytoplasm. RIG-I binds preferentially to short dsRNA with a 5'triphosphate [14]. MDA5 also recognizes dsRNA but seems to be more specific for higher molecular weight RNA fragments [15]. The third member of the family, LGP2, is not able to induce IFN despite having RNA-binding activity and it is believed to act as a regulator of RIG-I- and MDA5-mediated signaling [16]. Molecular pattern recognition by RIG-I or MDA5 triggers their interaction with the mitochondrial antiviral signaling protein (MAVS, also known as IPS-1, Cardif, and VISA), which will activate TRAF/TRADD and subsequently IRF3/IRF7 and NFκB transcription factors. Despite their recent discovery [17], RLR agonists have already been explored as antivirals [3], [18], [19] and as vaccine adjuvants [20], but to a far lesser extent than their TLR counterparts.

Little is known about the pathways involved in the detection of cytoplasmic DNA (see [21]–[23] for a complete review). The DNA receptors known until now are structurally far more heterogeneous than the TLR or RLR families. These same sensors are thought to be involved in the detection of endogenous nucleic acid in response to DNA damage or alterations in DNA clearance after uncontrolled cell death and also in the recognition of DNA from invading pathogens. So far, a common adapter for those receptors has not been found but all DNA sensing pathways seem to converge in the activation of IRF-3 as the transcription factor required for the production of IFN.

**Figure 2:**
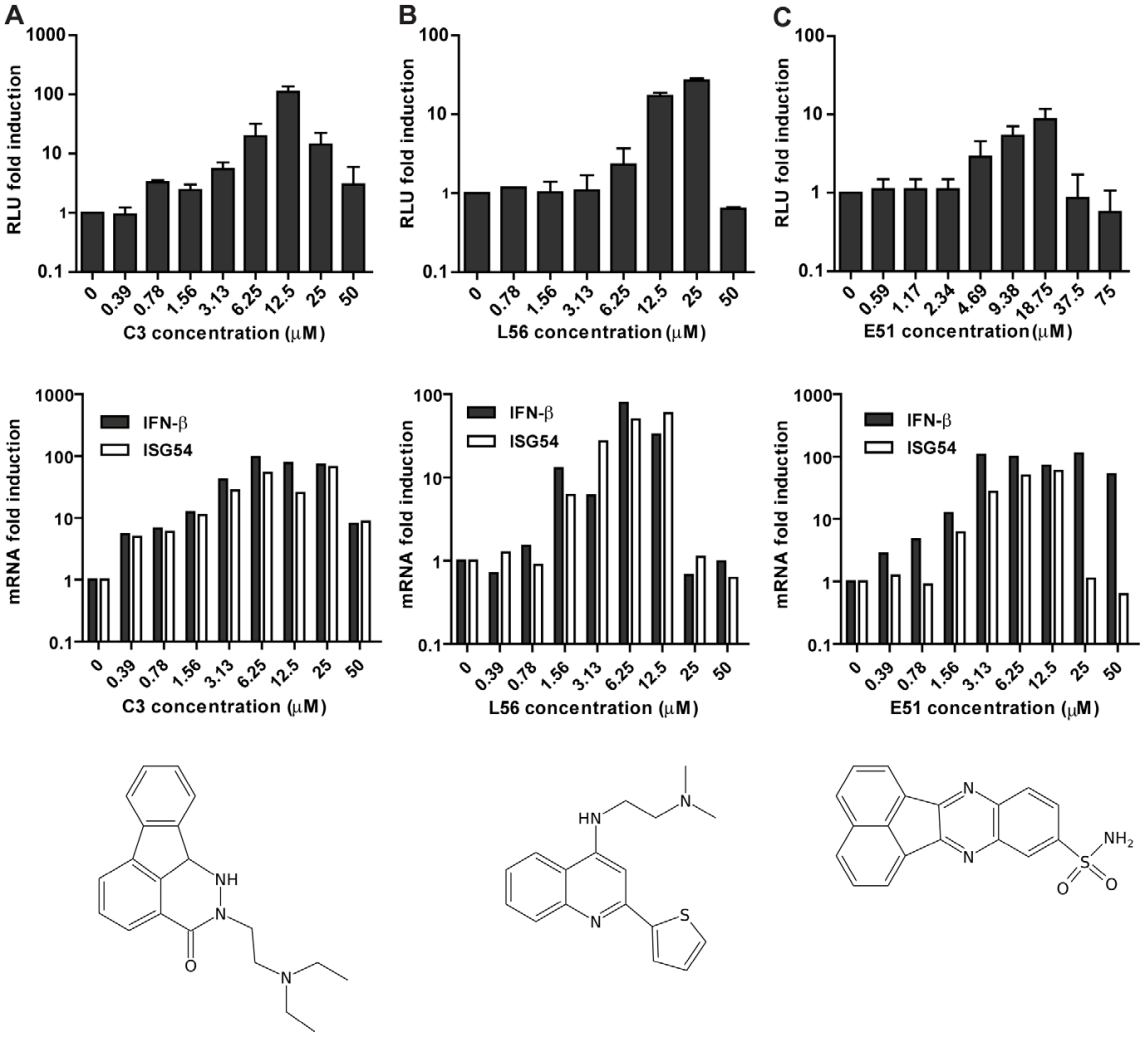
Activation of the 293T IFN reporter cell line by small molecules. **Top.** Luciferase fold induction of 293T-FF cells after 24 hrs treatment with C3 (**A**), L56 (**B**) or E51 (**C**) over the mock-treated cells. **Middle**. IFN-β (black bars) and ISG54 (white bars) mRNA induction in A549 cells after 24 hrs incubation with C3 (**A**), L56 (**B**) or E51 over the mock-treated cells. **Bottom**. Chemical structures of the small molecules, **A.** C3 3-[2-(diethylamino)ethyl]-2,3-diazatetracyclo[7.6.1.0?{5,16}.0?{10,15}]hexadeca-1,5(16),6,8,10,12,14-heptaen-4-one, **B.** L56 (N1,N1-dimethyl-N2-[2-(2-thienyl)-4-quinolyl]-1,2-ethanediamine) and **C.** E51 3,10-diazapentacyclo [10.7.1.0?{2,11}.0?{4,9}.0?{16,20}]icosa-1(19),2,4,6,8,10,12,14,16(20),17-decaene-6-sulfonamide.

Our objective is to find a drug-like molecule capable of stimulating the synthesis of IFN. In the present work, we describe our efforts to establish a simple and powerful methodology to screen, in a human cell-based, high throughput format, small molecules with the ability to induce IFN. In addition, we describe a lead molecule (C3) capable of inducing IFN and establishing an antiviral state *in vitro*, whose activity is not inhibited by the viral IFN antagonist proteins, influenza A virus NS1 or hepatitis C virus NS3/4A protein.

## Materials and Methods

### Cell Lines

293T, VERO and A549 cells were obtained from ATCC (http://www.atcc.org) and were maintained in Minimal Essential Medium (MEM) or Dulbecco's Modified Eagle Medium (DMEM) (Gibco, http://www.lifetechnologies.com) supplemented with 10% fetal bovine serum (FBS, Hyclone) and penicillin/streptomycin (Gibco). Primary hTBE cells (Lonza, http://www.lonza.com) were cultured in Bronchial Epithelial Cell Growth Medium supplemented with the BEGM SingleQuot kit (Lonza).

For the generation of a stable 293T cell line expressing firefly luciferase under the control of the IFN-β promoter (293T-FF) the plasmid pGL4.17-IFN-FF, encoding a cassette with the firefly luciferase gene under the control of the murine IFN-β promoter [24], was used to stably transfect the previously described 293T-ISRE-mRFP cells [25] in order to generate a double-reporter 293T-IFN-FF cell line. Transfected cells were selected in the presence of Geneticin (Invitrogen, http://www.invitrogen.com) and individual clones were isolated and tested for reporter induction upon Sendai virus infection. The selected clone was maintained in DMEM supplemented with 10% fetal bovine serum, 1% penicillin-streptomycin and 2 mg/mL of Geneticin.

**Figure 3:**
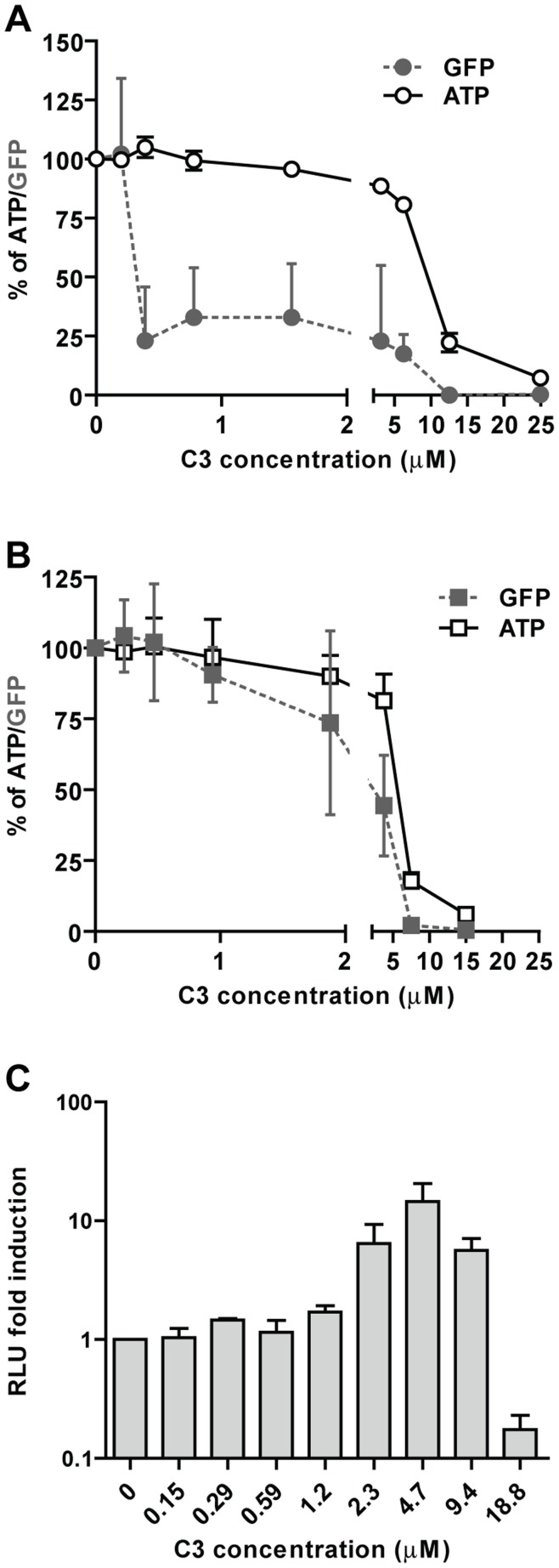
Induction of type I IFN by C3. To confirm the production of type I IFN A549 (**A**) or VERO (**B**) cells were incubated with increasing concentrations of C3 for 24 hrs, the media with the compound was then removed, the cells washed with PBS (2×) and new media containing VSV-GFP was added. Twenty-four hrs later the GFP (as an indirect estimation of the viral replication, dashed gray line) and the ATP (as marker for the cell viability, solid line) were measured. ATP and GFP values in non-treated cells were set to 100%. Error bars represent standard deviation of three replicates. **C.** C3 induction of luciferase in the IFN-β MDCK reporter cell line. MDCK cells expressing firefly luciferase under the control of the IFN-β promoter were treated with C3 at the indicated concentration. After 24 hrs the luciferase signal was analyzed and standardized based on the values obtained in the mock treated cells.

### Antiviral Bioassay

To test the biological activity of the IFN released by 293T-FF cells after SeV infection an antiviral bioassay was performed as previously described [26]. Briefly, 293T-FF cells were plated into 96-well plates and incubated at 37°C, 5% CO_2_ for 24 hours. The culture media was then replaced with post-infection media containing increasing amounts of SeV Cantell. At 24 hours post infection, supernatants were harvested and the virus present in the media was UV inactivated by placing the 96-well plate in a UV chamber (200 J/cm^2^). Inactivated supernatants were then added to fresh VERO cells previously seeded in 96-well plates. As a positive control we used media containing universal IFN (PBL Interferon Source, http://www.interferonsource.com). Following a 24 hour incubation, VERO cell supernatants were aspirated, and cells were infected with VSV-GFP diluted in OptiMEM. (The concentration of VSV-GFP used was previously determined to yield 90% GFP expression at 24 hours). GFP signal was visualized 24 hours post infection by fluorescence microscopy and quantified using a Beckman (Beckman Coulter, https://www.beckmancoulter.com) plate reader.

**Figure 4:**
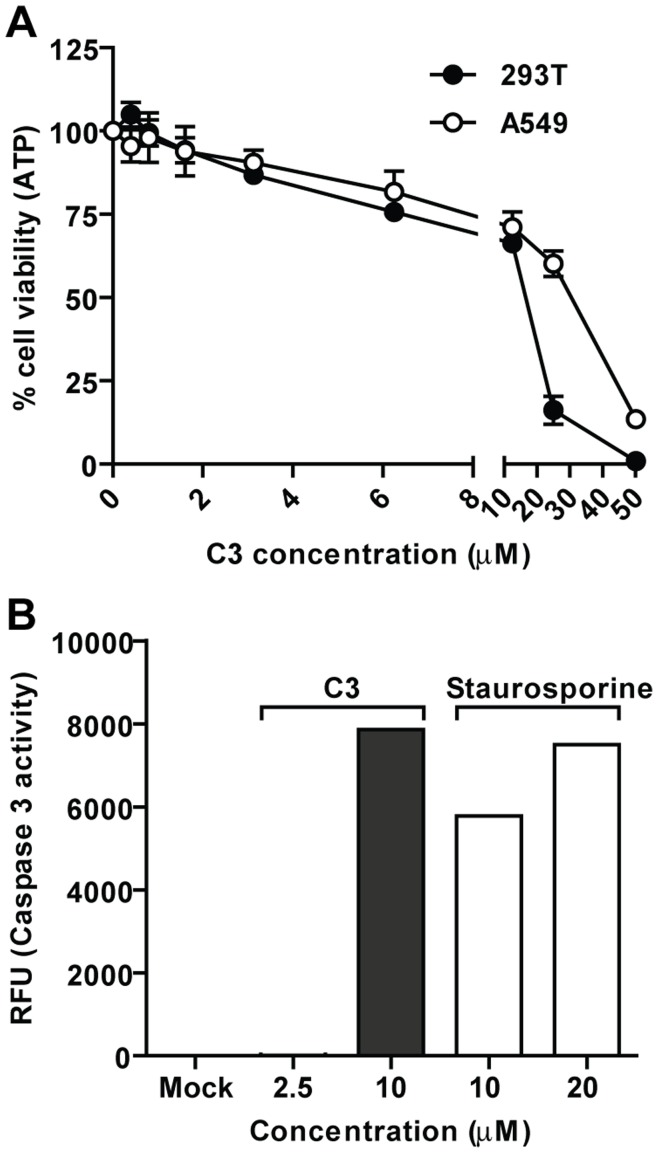
Cytotoxicity and apoptosis induction by C3. A. Percentage of ATP (representative of cell viability) in 293T-FF (black dots) and A549 (white dots) cells after 24 hrs incubation with C3 at increasing concentrations. The amount of ATP in mock-treated cells was set to 100%. Error bars represent standard deviation of three replicates. **B.** *In vitro* determination of caspase 3 activity by fluorimetric immunosorbent enzyme assay (Roche) after 24 hrs of C3 or Staurosporine treatment at the indicated concentrations.

### Small Molecular Weight Compounds and High-throughput Screening Assay

All compounds were provided by and screened at the National Screening Laboratory for the Regional Centers of Excellence in Biodefense and Emerging Infectious Diseases (NSRB) (Harvard Medical School, Boston, MA). The chemical libraries were stored at −20°C in desiccated storage containers.

To evaluate the robustness of our assays we calculated the Z'-factor [27], S/B ratio, and S/N ratio. Z' = 1-((3δ_pos_+3δ_neg_)/(µ_pos_- µ_neg_)), where µ_pos_ is the mean signal for the positive control, µ_neg_ is the mean signal for the negative control, δ_pos_ is the standard deviation of the positive control, and δ_neg_ is the standard deviation for the negative control. S/B = µ_pos_/µ_neg_. S/N = (µ_pos_- µ_neg_)/((δ_pos_)?2+(δ_neg_)?2)?1/2.

The HTS was performed in duplicate using Costar solid white 384-well plates. Plates were seeded, using an automated plate filler, with 35 µL of DMEM with 10% FBS containing 2×10^3^ 293T-FF cells per well, and cultured at 37°C, 5% CO_2_ for 12–14 hours. Compounds (100 nL) were then added using the Epson pin transfer robot (Epson, http://www.epson.com/). Two columns on the right side of each plate were reserved for controls and did not contain compounds. The second to last column was treated only with DMSO (negative control) and the last column was infected with Sendai virus (positive control). After 24 h at 37°C with 5% CO_2_ 35 µL of the Bright-Glo reagent (Promega, http://www.promega.com) diluted 3 fold with PBS, was added to each well and the luminescent signal was measured using an EnVision plate reader (Perkin Elmer, http://www.perkinelmer.com).

**Figure 5:**
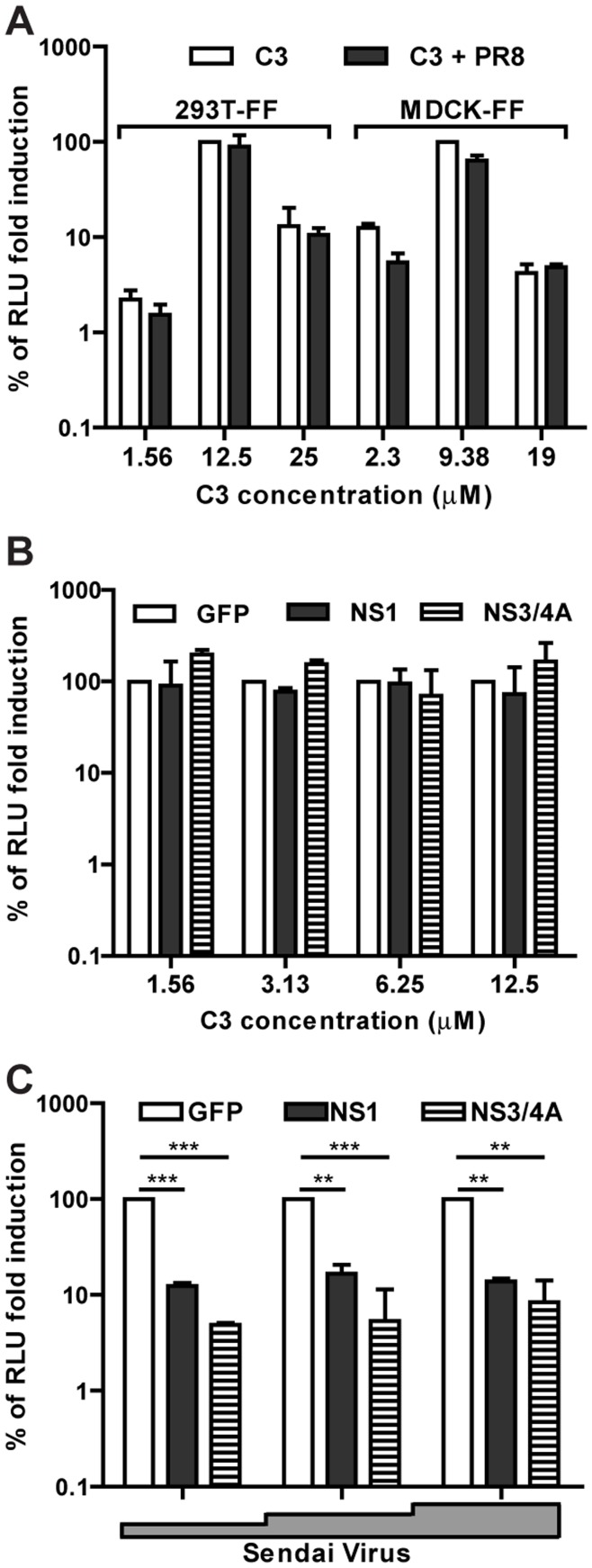
IFN-β induction by C3 in the presence of viral antagonist, NS1 and NS3/4A. **A** Induction of IFN by C3 in the absence (white bars) and presence (dark bars) of influenza A/PR/8/34 virus. Cells (either 293T-FF or MDCK expressing the firefly luciferase under the control of the IFN-β promoter) were infected with influenza A/PR/8/34 virus at an MOI of 10 and 2 hrs later treated with C3 at the indicated concentrations. Twenty hrs post-treatment the luciferase activity in the cells was measured as an indirect read-out of the IFN levels. The maximum luciferase value obtained from the mock infected cells for each cell line was set to 100%. **B.** and **C.** Induction of IFN in the presence of plasmid expressed NS1 from influenza A/PR/8/34 virus or NS3/4A from HCV. 293T-FF cells were transfected with either GFP (white bars), NS1 (dark bars) or NS3/4A (hatched bars), 24 hrs later the media was removed and fresh media containing C3 (C) or Sendai Virus (D) added. The luciferase levels were measured 24 hrs later and the relative luminescence units (RLU) of the GFP transfected cells set to 100%. Error bars represent standard deviation of three replicates. The significance of the differences was calculated using a t-test, ns indicates not significant, **pvalue<0.01 and ***pvalue<0.001.

Results obtained from the screen were standardized using the Z-Score, calculation which indicates how many standard deviations a particular compound is above or below the mean of the plate, and is calculated as follows: Z-Score = (x-μ)/δ, where x is the raw signal, μ is the mean signal of all the compound-containing wells of one plate, and δ is the standard deviation of all compound-containing wells of one plate. Primary hits were identified by calculating a Z-score for each compound and applying hit selection criteria: A compound was considered a hit if one replicate had a Z-Score >2.9 and the other replicate had a Z-Score of >2.

UIPAC names of the selected hits: C3 3-[2-(diethylamino)ethyl]-2,3-diazatetracyclo[7.6.1.0?{5,16}.0?{10,15}]hexadeca-1,5(16),6,8,10,12,14-heptaen-4-one. E51 3,10-diazapentacyclo[10.7.1.0?{2,11}.0?{4,9}.0?{16,20}]icosa-1(19),2,4,6,8,10,12,14,16(20),17-decaene-6-sulfonamide. L56 N1,N1-dimethyl-N2-[2-(2-thienyl)-4-quinolyl]-1,2-ethanediamine.

SMILES:C3 CCN(CC)CCn1nc2-c3ccccc3-c3cccc(c23)c1 = O, E51 NS( = O)( = O)c1ccc2nc-3c(nc2c1)-c1cccc2cccc-3c12 and L56 CN(C)CCNc1cc(nc2ccccc12)c1cccs1.

**Figure 6:**
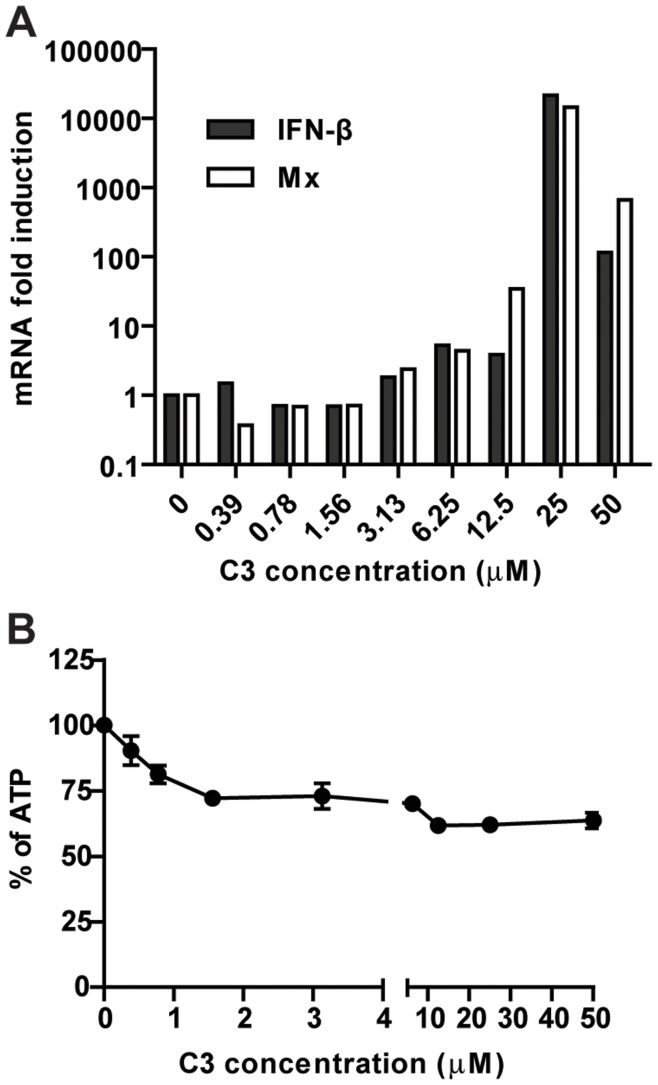
Induction of IFN and ISGs by C3 in hTBE cells. **A.** IFN-β (black bars) and Mx (white bars) mRNA induction in primary human tracheobronchial epithelial (hTBE) cells after 24 hrs incubation with C3. **B.** Percentage of ATP (representative of cell viability) in hTBE cells after 24 hrs incubation with C3 at increasing concentrations. The amount of ATP in mock-treated cells was set to 100%. Error bars represent standard deviation of three replicates.

### Cell Viability Assay and Caspase 3 Activity Assay

The CellTiterGlo Cell Viability Assay (Promega, http://www.promega.com) was used to detect ATP levels, as a function of cell viability, according to manufacturer's specifications. Cells were seeded into 96-well plates (1500–2000 cells/well) and incubated at 37°C, 5% CO2 for 24 hours. Culture medium was then replaced with 50 µL of fresh medium containing compound (serially diluted), and further incubated for 24 h hours. The final concentration of DMSO in the culture medium did not exceed 0.5%. Luciferase production was then measured by adding 50 µL of CellTiterGlo reagent (diluted 2 fold with PBS) to each well, and the luminescence signal read using the Beckman plate reader. Caspase 3 activity was measured using the caspase 3 Activity Assay from Roche (www.roche.com) according to the manufacturer's protocol at 24 hrs post-treatment with C3. Staurosporine (Sigma-Aldrich, http://www.sigmaaldrich.com) was used as a positive control in the caspase 3 activity assay.

### RNA Extraction and qRT-PCR

RNA from mammalian cell culture was obtained using the RNeasy kit from Qiagen (www.qiagen.com). Residual DNA in the samples was removed using DNA-free (Ambion). cDNA was synthesized using SuperScript® III Reverse Transcriptase (Invitrogen, http://www.invitrogen.com) following the manufacturer's instructions. Real-time PCR quantification was performed using SYBR Green mix (Roche, http://www.roche.com) and a LightCycler 480 Instrument (Roche). Samples were normalized to β-actin. Primers sequences for qRT-PCR are available upon request.

## Results

### Generation of an Interferon Reporter Human Cell Line

For identification of small molecules that activate IFN-β production, we created a reporter 293T reporter cell line that stably expresses the firefly luciferase under the control of the IFN-β promoter [28]. We intentionally selected 293T cells because of their human origin and their deficiency in the expression of most TLRs [29]. This choice therefore biased our screen towards compounds that do not require TLRs for their IFN-inducing activity.

Stimulation of the 293T IFN-β reporter cell line (293T-FF) with increasing amounts of Sendai virus Cantell (SeV), a well known inducer of IFN via RIG-I [14], [30], resulted in increasing luciferase signal (Figure 1A). However stimulation with the TLR4 agonist LPS, as expected, does not induce luminescence (Figure 1A). To corroborate that the levels of luciferase correlate with production of IFN-β we collected the supernatants from 293T-FF cells previously infected with SeV and performed a bioassay in Type I IFN-deficient VERO cells [31], [32]. Briefly, VERO cells were cultured for 24 hours (hrs) with either UV-inactivated supernatant from 293T-FF SeV infected cells or media supplemented with universal IFN as a control. Cells were then infected with a vesicular stomatitis virus expressing the green fluorescent protein (VSV-GFP) for an additional 24 hrs, after which the fluorescence was measured. The presence of IFN in the media will stimulate an anti-viral state in the cells and will therefore attenuate or inhibit viral growth. The intensity of the GFP signal (an indirect estimation of VSV replication) will consequently be inversely proportional to the amount of IFN in the supernatants. The result of the experiment (Figure 1B) clearly indicated a direct correlation between firefly luciferase signal and type I IFN production in 293T-FF cells. This result was further confirmed by measuring the IFN-β mRNA and IFN-β secreted protein produced by 293T-FF after SeV stimulation (Figure 1C). Consequently the firefly luciferase signal produced by the 293T-FF cells faithfully represents the production of IFN-β. This allowed us to use the modified cell line as a reporter in a high-throughput screen looking for IFN-inducing small molecules.

### Identification of IFN Inducing Compounds

For the identification of small molecules with IFN-inducing properties in a high-throughput screen (HTS), we developed a simple and yet robust assay. The protocol was first optimized in a 96-well format and then miniaturized and re-optimized in a 384-well format. We verified its suitability for use in a HTS by the Z' factor (Z' = 0.34), signal-to-noise ratio (S/N = 4.57) and the signal-to-background ratio (S/B = 11,934.61). For the screening, 293T-FF cells were seeded in complete media and 12–14 hrs later infected with SeV (positive control), treated with DMSO (negative control), or treated with the appropriate small molecule. After 24 hrs luminescence was measured and hits were selected on an increased signal over the solvent-treated cells.

A library consisting of 94,398 structurally diverse small molecules was screened in duplicate at the National Screening Laboratory for the Regional Centers of Excellence in Biodefense and Emerging Infectious Diseases (NSRB) (Harvard Medical School). The results were standardized by the Z-Score and hits selected based on minimum Z-Scores of 2.9 and 2 from duplicate samples. We identified 97 hits, which represent a hit rate of 0.102%. At this point we included 24 additional hits that came from a previous screen performed in influenza virus infected Madin Darby canine kidney cells (MDCK) that also stably carry the firefly luciferase gene under control of IFN-β reporter [33], making a total of 121 compounds.

To confirm the hits from the HTS we first tested the candidate small molecules in a dose-response assay using the 293T-FF cells. The compounds were confirmed as a hit only if a dose-dependent induction of luciferase was observed with at least one point having a fold induction over the mock treated cells greater than 5. Along with the induction of IFN we also analyzed the toxicity of these compounds in the reporter cell line. Based on these results a subset of compounds was selected and their ability to induce IFN-β and ISG54 mRNA in human lung epithelial derived cells (A549) along with their toxicity in this cell line, was determined. We next tested the induction of IFN in canine (using the IFN-reporter cells used in [33]) and in murine cells (by qRT-PCR), looking for compounds that can stimulated the innate immune system across species. Three small molecules (C3, L56 and E51) were able to induce IFN in human, canine and murine cell lines, while 15 compounds were human-specific IFN-inducers. Results on reporter gene induction on 293T-FF cells, induction of IFN-β and Mx mRNA and chemical structures for the three compounds are shown in Figure 2. Due to its greater potency (Figure 2) and its cross-species activity C3 was selected as our lead candidate.

### C3 Stimulates the Production of Type I Interferon

C3 induces in a dose dependent manner synthesis of firefly luciferase in the IFN-β reporter 293T (Figure 2A) and MDCK cell lines (Figure 3C). C3 also stimulates IFN-β and ISG54 mRNA production in A549 cells (Figure 2A) and IFN-β and Mx mRNA in mouse embryonic fibroblast (data not shown). If C3 is indeed capable of inducing IFN-β at non-toxic concentrations it should also promote an antiviral state in the host cell. To test this hypothesis we treated A549 cells with C3 at different doses. After 24 hrs, the media with the compound was removed, the cells washed and new media containing VSV-GFP was added. Approximately 24 hrs after the infection, we measured green fluorescence, as an indirect read-out of VSV replication, and ATP levels to monitor the toxicity of the small molecule. In cells pre-treated with C3, at concentration where no significant toxicity was observed, we detected reduced VSV-GFP replication (Figure 3A). To verify that this protection was in fact due to the induction of type I IFN, we performed the same assay in IFN-deficient VERO cells [31], [32]. In this case GFP levels were not significantly reduced (Figure 3B), confirming that the reduction in GFP signal seen in A549 was primarily due to the antiviral state achieved by the cells after exposure to C3-induced type I IFN.

The toxicity profile in both 293T-FF and A549 cells is very similar and shows relatively low toxicity below 10 µM with a drop in cell viability around 20–40 µM (Figure 4A). Over-stimulation of IFN production has been correlated with induction of apoptosis [34]. In fact, the intense cross-talk between these two processes is reflected in the large number of proteins involved in both mechanisms [35]–[38]. We therefore considered whether this abrupt increase in cell death could be due to activation of apoptosis. To test this hypothesis, we measured the caspase 3 activity in mock treated cells versus cells treated with C3 at different concentrations over 24 hrs. The experiment also included staurosporine as a positive control for apoptosis induction. No caspase 3 activity was detected in the cells treated with C3 at 2.5 µM (concentration where the cell viability was above 80%). At 10 µM however, the levels of caspase 3 activity were similar to those observed when the cells were treated with staurosporine (Figure 4B) indicating induction of apoptosis. It is important to note that not all apoptosis activators have the ability to induce IFN. Using our reporter cell line we tested staurosporine for IFN induction and found that no luciferase production was observed (data not shown).

C3 could only be used as an antiviral if its IFN stimulatory effects are not adversely affected by the presence of viral infection, in particular the presence of viral antagonists of the IFN pathway. In the case of influenza virus the major viral suppressor of IFN is the non-structural protein 1 (NS1). NS1 is capable of blocking the activation of RIG-I [39] and thereby preventing the induction of IFN. Infection of either 293T-FF or MDCK-FF cells with influenza A/PR8/34 virus did not reduce the IFN stimulating properties of C3 (Figure 5A), leading to the conclusion that the presence of NS1 protein, despite its IFN antagonist role, does not block C3 activity. To confirm this result 293T-FF cells were transfected with influenza A/WSN/33 virus NS1 protein and stimulated with C3 24 hrs later. Once more, C3 IFN-inducing activity was no reduced by the presence of NS1 (Figure 5B). We also included SeV as a stimulus, and as IFN induction by SeV is highly dependent on RIG-I, we observed diminished signal in the presence of NS1 (Figure 5C). Downstream of RIG-I is the signal adaptor MAVS. Hepatitis C virus (HCV) carries an IFN suppressor (NS3/4A) that proteolytically cleaves MAVS thus preventing any downstream signaling [40]. Transfection of a plasmid expressing NS3/4A did not block C3 induction of IFN. On the other hand, SeV IFN induction was again inhibited by the viral IFN antagonist.

Finally to assess the IFN-inducing properties of C3 in a more relevant system primary human tracheobronchial epithelial cells (hTBE) were treated with C3 at different doses. C3 was capable of inducing IFN-β and the IFN stimulated gene Mx (Figure 6B). Furthermore the compound induced toxicity observed was lower in hTBE cells than in A549 or 293T cells (Figure 6C).

## Discussion

Type I IFN participates in the regulation of both innate and adaptive immune responses. Once it is produced this family of cytokines acts in an autocrine and paracrine manner changing the expression profile of multiple genes. These IFN regulated genes will lead, initially to the induction of a non-specific anti-viral state to halt virus replication [41], and ultimately to cell arrest or even apoptosis to control the ongoing infection [42], [43]. IFN also promotes activation of dendritic cells, T cells, B cells and NK cells enhancing the adaptive immune response against the invading pathogen [7]. IFN can therefore potentially be used as a broad-spectrum antiviral, as a vaccine adjuvant and also for cancer treatment. Despite their promising properties, the clinical application of IFN or IFN-inducing molecules (e.g. poly I:C) has been limited so far. This is in part due to the nature/structure of the molecules being used, which in many cases is not suitable for clinical applications. Our objective is to obtain a drug-like small molecule candidate with IFN-inducing properties that could potentially overcome these hurdles. The identification of a compound with these characteristics has receive a lot of attention. In fact, recently, other groups have reported small-molecules capable of promoting a cellular antiviral state by activation of the innate immune system [44], [45].

To be able to screen for small molecules that induce IFN, we first created a human derived reporter cell line that expresses firefly luciferase under control of the IFN-β promoter. Our results show a direct correlation between the firefly luciferase and the endogenous IFN-β produced, which together with the reproducibility of the response allows for a fast and reliable identification of compounds that induce type I IFN. We were then able to establish an HTS assay for the identification of IFN-inducing small molecules in a medium and high-throughput format. Using this protocol we screened a library of 94,398 compounds, among which we found 3 small molecules capable of inducing IFN in human, canine and murine derived cells: C3, E51 and L56.

In human cells C3 generates a dose-dependent induction of IFN that peaks, depending on the cell line, around 10 µM. Above that concentration the toxicity of the compound increases rapidly. The caspase 3 activity indicates that apoptosis is activated only at high concentrations, which correlates with the toxicity profile and is likely the reason for the sudden decline in cell viability, although other reasons can not be ruled out at this point.

At sub-toxic concentrations, C3 induces significant amounts of IFN. Treatment with C3 promotes an antiviral state in A549 that protects them from VSV-GFP infection. Protection was not observed when using type I IFN incompetent VERO cells [31], [32], but a small reduction of VSV-GFP replication around 4 µM, which could be attributed to induction, in this cell line, of other cytokines or chemokines beside type I IFN. This result demonstrates the IFN inducing and antiviral properties of C3 *in vitro.* Unfortunately C3 has a very short half life of approximately 2–3 minutes in murine liver microsomes (data not shown), suggesting that is metabolically unstable. We should therefore obtain a more stable analog via medicinal chemistry before we attempt any *in vivo* experiments.

The exact mechanism by how C3 induces IFN is still undetermined. However, C3 maintains its IFN inducing activity in the presence of influenza A virus NS1 or hepatitis C virus NS3/4A proteins, which clearly indicates that C3 does not utilize RIG-I or MAVS to induce IFN. The RLR/MAVS pathway is fundamental to the recognition of a wide variety of RNA viruses, and in fact it is targeted for inhibition by many viruses including influenza A virus, HCV, RSV and Ebola virus (see [46] for a full review). A small molecule that can “release” the viral restriction on the production of IFN by stimulating its production through another pathway or activating a component downstream of the targets of NS1 and NS3 could potentially be used as a broad spectrum antiviral.

In our HTS for IFN-inducing compounds we also identified several known bioactives small molecules such as doxorubicin, aminacrine, ellipticine and aklavine that were also, confirmed in our secondary assays as a IFN inducers (data not shown). These molecules are DNA intercalators used as anticancer drugs or as antibiotics. By searching the literature for studies on compounds structurally similar to C3 we discovered that it was previously identified in a HTS as an antibacterial agent [47] and later suggested to be a mitochondrial type II topoisomerase inhibitor with the ability to unwind DNA [48]. The DNA binding activity of C3 would suggest a similar mechanism of action for its IFN induction to that proposed for other FDA approved topoisomerase inhibitors such as doxorubicin, which involves recognition of the compound induced DNA stress and IRF3 nuclear translocation [49], [50]. More investigation will be required to determine whether this is the case. Our results expand the knowledge on C3-like molecules and suggest novel uses for these compounds. The emergence of viral resistance against current anti-viral drugs [51], [52] and the lack of effective antivirals for several viral disease provides a rationale for the development of new broad-spectrum antivirals. C3 may represent a starting point for the development of broad-spectrum antivirals and possibly also vaccine adjuvants that act by promoting IFN induction. However, more *in vivo* experiments (including efficacy and safety studies), with a more stable analog, are required to determine the true potential of C3-like molecules.

## References

[1] Keam SJ, Cvetković RS (2008) Peginterferon-alpha-2a (40 kd) plus ribavirin: a review of its use in the management of chronic hepatitis c mono-infection. Drugs 68: 1273–1317. 18547135 10.2165/00003495-200868090-00006

[2] Bergman SJ, Ferguson MC, Santanello C (2011) Interferons as therapeutic agents for infectious diseases. Infect Dis Clin North Am 25: 819–834. 22054758 10.1016/j.idc.2011.07.008 PMC7134994

[3] Falcoff E, Falcoff R, Cherby J, Florent J, Lunel J, et al (1973) Double-stranded ribonucleic acid from mengo virus: production, characterization, and interferon-inducing and antiviral activities in comparison with polyriboinosinic-polyribocytidylic acid. Antimicrob Agents Chemother 3: 590–598. 4364180 10.1128/aac.3.5.590 PMC444463

[4] Field AK, Tytell AA, Lampson GP, Hilleman MR (1967) Inducers of interferon and host resistance. ii. multistranded synthetic polynucleotide complexes. Proc Natl Acad Sci U S A 58: 1004–1010. 5233831 10.1073/pnas.58.3.1004 PMC335739

[5] Panter G, Kuznik A, Jerala R (2009) Therapeutic applications of nucleic acids as ligands for toll-like receptors. Curr Opin Mol Ther 11: 133–145. 19330719

[6] Miller RL, Meng T, Tomai MA (2008) The antiviral activity of toll-like receptor 7 and 7/8 agonists. Drug News Perspect 21: 69–87. 18389099 10.1358/dnp.2008.21.2.1188193

[7] Toporovski R, Morrow MP, Weiner DB (2010) Interferons as potential adjuvants in prophylactic vaccines. Expert Opin Biol Ther 10: 1489–1500. 20836750 10.1517/14712598.2010.521495

[8] Rizza P, Capone I, Moretti F, Proietti E, Belardelli F (2011) Ifn-α as a vaccine adjuvant: recent insights into the mechanisms and perspectives for its clinical use. Expert Rev Vaccines 10: 487–498. 21506646 10.1586/erv.11.9

[9] Prchal M, Pilz A, Simma O, Lingnau K, von Gabain A, et al (2009) Type i interferons as mediators of immune adjuvants for t- and b cell-dependent acquired immunity. Vaccine 27 Suppl 6G17–20. 20006134 10.1016/j.vaccine.2009.10.016

[10] Tovey MG, Lallemand C (2010) Adjuvant activity of cytokines. Methods Mol Biol 626: 287–309. 20099135 10.1007/978-1-60761-585-9_19

[11] Kuznik A, Panter G, Jerala R (2010) Recognition of nucleic acids by toll-like receptors and development of immunomodulatory drugs. Curr Med Chem 17: 1899–1914. 20377514 10.2174/092986710791163957

[12] Martinez-Martin N, Viejo-Borbolla A (2010) Toll-like receptor-mediated recognition of herpes simplex virus. Front Biosci (Schol Ed) 2: 718–729. 20036979 10.2741/s96

[13] Klein Klouwenberg P, Tan L, Werkman W, van Bleek GM, Coenjaerts F (2009) The role of toll-like receptors in regulating the immune response against respiratory syncytial virus. Crit Rev Immunol 29: 531–550. 20121698 10.1615/critrevimmunol.v29.i6.40

[14] Baum A, Sachidanandam R, García-Sastre A (2010) Preference of rig-i for short viral rna molecules in infected cells revealed by next-generation sequencing. Proc Natl Acad Sci U S A 107: 16303–16308. 20805493 10.1073/pnas.1005077107 PMC2941304

[15] Loo Y, Gale MJ (2011) Immune signaling by rig-i-like receptors. Immunity 34: 680–692. 21616437 10.1016/j.immuni.2011.05.003 PMC3177755

[16] Kumar H, Kawai T, Akira S (2011) Pathogen recognition by the innate immune system. Int Rev Immunol 30: 16–34. 21235323 10.3109/08830185.2010.529976

[17] Yoneyama M, Kikuchi M, Natsukawa T, Shinobu N, Imaizumi T, et al (2004) The rna helicase rig-i has an essential function in double-stranded rna-induced innate antiviral responses. Nat Immunol 5: 730–737. 15208624 10.1038/ni1087

[18] Miller DK, Lenard J (1982) Ultraviolet-irradiated vesicular stomatitis virus and defective-interfering particles are similar non-specific inhibitors of virus infection. J Gen Virol 60: 327–333. 6286855 10.1099/0022-1317-60-2-327

[19] Wong JP, Saravolac EG, Sabuda D, Levy HB, Kende M (1995) Prophylactic and therapeutic efficacies of poly(ic.lc) against respiratory influenza a virus infection in mice. Antimicrob Agents Chemother 39: 2574–2576. 8585749 10.1128/aac.39.11.2574 PMC162988

[20] Herniman KA, Sellers RF (1972) Protection of guinea-pigs against foot-and-mouth disease by simultaneous inoculation of sendai virus and inactivated foot-and-mouth disease vaccine. Arch Gesamte Virusforsch 37: 97–103. 4336969 10.1007/BF01241155

[21] Nagarajan U (2011) Induction and function of ifnβ during viral and bacterial infection. Crit Rev Immunol 31: 459–474. 22321107 10.1615/critrevimmunol.v31.i6.20 PMC3281552

[22] Barber GN (2011) Innate immune dna sensing pathways: sting, aimii and the regulation of interferon production and inflammatory responses. Curr Opin Immunol 23: 10–20. 21239155 10.1016/j.coi.2010.12.015 PMC3881186

[23] Cavlar T, Ablasser A, Hornung V (2012) Induction of type i ifns by intracellular dna-sensing pathways. Immunol Cell Biol 90(5): 474–82. 22450802 10.1038/icb.2012.11

[24] Hai R, Martínez-Sobrido L, Fraser KA, Ayllon J, García-Sastre A, et al (2008) Influenza b virus ns1-truncated mutants: live-attenuated vaccine approach. J Virol 82: 10580–10590. 18768976 10.1128/JVI.01213-08 PMC2573209

[25] Nguyen DN, Kim P, Martínez-Sobrido L, Beitzel B, García-Sastre A, et al (2009) A novel high-throughput cell-based method for integrated quantification of type i interferons and in vitro screening of immunostimulatory rna drug delivery. Biotechnol Bioeng 103: 664–675. 19338049 10.1002/bit.22312 PMC2771114

[26] Hoffmann H, Kunz A, Simon VA, Palese P, Shaw ML (2011) Broad-spectrum antiviral that interferes with de novo pyrimidine biosynthesis. Proc Natl Acad Sci U S A 108: 5777–5782. 21436031 10.1073/pnas.1101143108 PMC3078400

[27] Zhang J, Chung T, Oldenburg K (1999) A simple statistical parameter for use in evaluation and validation of high throughput screening assays. J Biomol Screen 4: 67–73. 10838414 10.1177/108705719900400206

[28] Martínez-Sobrido L, Zúñiga EI, Rosario D, García-Sastre A, de la Torre JC (2006) Inhibition of the type i interferon response by the nucleoprotein of the prototypic arenavirus lymphocytic choriomeningitis virus. J Virol 80: 9192–9199. 16940530 10.1128/JVI.00555-06 PMC1563941

[29] Kirschning CJ, Wesche H, Merrill Ayres T, Rothe M (1998) Human toll-like receptor 2 confers responsiveness to bacterial lipopolysaccharide. J Exp Med 188: 2091–2097. 9841923 10.1084/jem.188.11.2091 PMC2212382

[30] Strahle L, Garcin D, Kolakofsky D (2006) Sendai virus defective-interfering genomes and the activation of interferon-beta. Virology 351: 101–111. 16631220 10.1016/j.virol.2006.03.022

[31] Desmyter J, Melnick JL, Rawls WE (1968) Defectiveness of interferon production and of rubella virus interference in a line of african green monkey kidney cells (vero). J Virol 2: 955–961. 4302013 10.1128/jvi.2.10.955-961.1968 PMC375423

[32] Emeny JM, Morgan MJ (1979) Regulation of the interferon system: evidence that vero cells have a genetic defect in interferon production. J Gen Virol 43: 247–252. 113494 10.1099/0022-1317-43-1-247

[33] Ortigoza MB, Dibben O, Maamary J, Martinez-Gil L, Leyva-Grado VH, et al (2012) A novel small molecule inhibitor of influenza a viruses that targets polymerase function and indirectly induces interferon. PLoS Pathog 8: e1002668. 22577360 10.1371/journal.ppat.1002668 PMC3343121

[34] Meyer O (2009) Interferons and autoimmune disorders. Joint Bone Spine 76: 464–473. 19773191 10.1016/j.jbspin.2009.03.012

[35] Cheng Y, Xu F (2011) Anticancer function of polyinosinic-polycytidylic acid. Cancer Biol Ther 10: 1219–1223. 10.4161/cbt.10.12.13450 20930504

[36] Burel SA, Machemer T, Ragone FL, Kato H, Cauntay P, et al.. (2012) Unique moe-dna chimeric oligonucleotide induces an atypical mda5 dependent induction of type i interferon response. J Pharmacol Exp Ther :. 10.1124/jpet.112.193789 22505629

[37] Liu F, Gu J (2011) Retinoic acid inducible gene-i, more than a virus sensor. Protein Cell 2: 351–357. 21626268 10.1007/s13238-011-1045-y PMC4875335

[38] Rayet B, Gélinas C (1999) Aberrant rel/nfkb genes and activity in human cancer. Oncogene 18: 6938–6947. 10602468 10.1038/sj.onc.1203221

[39] Hale BG, Albrecht RA, García-Sastre A (2010) Innate immune evasion strategies of influenza viruses. Future Microbiol 5: 23–41. 20020828 10.2217/fmb.09.108 PMC2820251

[40] Loo Y, Owen DM, Li K, Erickson AK, Johnson CL, et al (2006) Viral and therapeutic control of ifn-beta promoter stimulator 1 during hepatitis c virus infection. Proc Natl Acad Sci U S A 103: 6001–6006. 16585524 10.1073/pnas.0601523103 PMC1458687

[41] Wang BX, Fish EN (2012) The yin and yang of viruses and interferons. Trends Immunol 33: 190–197. 22321608 10.1016/j.it.2012.01.004 PMC7106503

[42] Inao T, Harashima N, Monma H, Okano S, Itakura M, et al (2011) Antitumor effects of cytoplasmic delivery of an innate adjuvant receptor ligand, poly(i:c), on human breast cancer. Breast Cancer Res Treat: 134 (1): 89–100. 10.1007/s10549-011-1930-3 22203435

[43] Tagawa M, Kawamura K, Li Q, Tada Y, Hiroshima K, et al (2011) A possible anticancer agent, type iii interferon, activates cell death pathways and produces antitumor effects. Clin Dev Immunol 2011: 479013. 22013482 10.1155/2011/479013 PMC3195555

[44] Bedard KM, Wang ML, Proll SC, Loo Y, Katze MG, et al (2012) Isoflavone agonists of irf-3 dependent signaling have antiviral activity against rna viruses. J Virol 86: 7334–7344. 22532686 10.1128/JVI.06867-11 PMC3416323

[45] Patel DA, Patel AC, Nolan WC, Zhang Y, Holtzman MJ (2012) High throughput screening for small molecule enhancers of the interferon signaling pathway to drive next-generation antiviral drug discovery. PLoS ONE 7: e36594. 22574190 10.1371/journal.pone.0036594 PMC3344904

[46] Versteeg GA, García-Sastre A (2010) Viral tricks to grid-lock the type i interferon system. Curr Opin Microbiol 13: 508–516. 20538505 10.1016/j.mib.2010.05.009 PMC2920345

[47] Cheng B, Liu I, Tse-Dinh Y (2007) Compounds with antibacterial activity that enhance dna cleavage by bacterial dna topoisomerase i. J Antimicrob Chemother 59: 640–645. 17317696 10.1093/jac/dkl556

[48] Tang SC, Shapiro TA (2010) Newly identified antibacterial compounds are topoisomerase poisons in african trypanosomes. Antimicrob Agents Chemother 54: 620–626. 20008775 10.1128/AAC.01025-09 PMC2812133

[49] Wheeler HR, Geczy CL (1990) Induction of macrophage procoagulant expression by cisplatin, daunorubicin and doxorubicin. Int J Cancer 46: 626–632. 2120134 10.1002/ijc.2910460413

[50] Kim T, Kim TY, Song YH, Min IM, Yim J, et al (1999) Activation of interferon regulatory factor 3 in response to dna-damaging agents. J Biol Chem 274: 30686–30689. 10521456 10.1074/jbc.274.43.30686

[51] Hurt AC, Chotpitayasunondh T, Cox NJ, Daniels R, Fry AM, et al (2012) Antiviral resistance during the 2009 influenza a h1n1 pandemic: public health, laboratory, and clinical perspectives. Lancet Infect Dis 12: 240–248. 22186145 10.1016/S1473-3099(11)70318-8

[52] Farci P (2011) New insights into the hcv quasispecies and compartmentalization. Semin Liver Dis 31: 356–374. 22189976 10.1055/s-0031-1297925

